# Supplementary material for: Content-rich biological network constructed by mining PubMed abstracts
Source: BMC Bioinformatics. 2004 Oct 8;5:147. doi: 10.1186/1471-2105-5-147 (PMC528731; doi:10.1186/1471-2105-5-147)
Supplement: Additional File 2 — The original results of the above study (non-essential files are deleted to keep the file size under the limit set by BMC bioinformatics). [file 1471-2105-5-147-S2.bz2 › chilibotAdditionalFile2/dip20/388ID11297552E9862/html/PDGFRA_PDGFB.html]

 


 **PDGFRA** and **PDGFB** 
  
Found 113 abstracts in PubMed, retrieved 20.  
 

 What does Google say? 
 PDF only 
| .edu only 

---

**Interactive relationship** (e.g. stimulation, inhibition, etc)

**Non-interactive relationship** (e.g. studied together, co-existance, homology, etc.)

- Content and activity of cAMP response element binding protein regulate platelet derived growth factor  [ **PDGFB** ]  receptor alpha  [ **PDGFRA** ]  content in vascular smooth muscles.  Ref: 12130557 Endocrinology, 2002
- Platelet derived growth factor  [ **PDGFB** ]  receptor A, insulin like growth factor 1 receptor, Janus kinase 1, TYK2, discoidin domain receptor 2, and Lyn were expressed in SF, and the expression of  **PDGFRA** , IGF 1R, and DDR2 in SF of RA were higher than that of osteoarthritis.  Ref: 12419287 J Autoimmun, 2002
- The Fgfr3 cells are interspersed with but distinct from platelet derived growth factor  [ **PDGFB** ]  receptor alpha  [ **PDGFRA** ]   **Pdgfra**  positive oligodendrocyte progenitors.  Ref: 12441294 Development, 2003
- Semi quantitativereverse transcription polymerase chain reaction was used to compare the relative abundance of 2 transcripts for housekeeping proteins beta actin and ribosomal protein L30 and 3 transcripts for growth factor ligand or receptors platelet derived growth factor  [ **PDGFB** ]  receptor alpha  [ **PDGFRA** ]  PDGFRalpha, basic fibroblast growth factor bFGF, in mature bovine oocytes of high versus low developmental potential.  Ref: 12760483 Can J Vet Res, 2003
- early oligodendroglial markers were chondroitin sulfate proteoglycan antigen and platelet derived growth factor  [ **PDGFB** ]  receptor alpha  [ **PDGFRA** ] .  Ref: 12271492 J Comp Neurol, 2002
- These included absence of expression of CD44, E NCAM, A2B5, epidermal growth factor receptor EGFR, and platelet derived growth factor  [ **PDGFB** ]  receptor alpha  [ **PDGFRA** ]  PDGFR alpha, suggesting that negative selection using cell surface epitopes could be used to isolate stem cell populations from mixed cultures of cells.  Ref: 12435354 Dev Biol, 2002
- Here we show that approximately 35% 14 of 40 of GISTs lacking KIT mutations have intragenic activation mutations in the related receptor tyrosine kinase, platelet derived growth factor  [ **PDGFB** ]  receptor alpha  [ **PDGFRA** ]   **PDGFRA** .  Ref: 12522257 Science, 2003
- We have previously shown that deregulated expression of the platelet derived growth factor  [ **PDGFB** ]  alpha receptor  **PDGFRA**  can be associated with neural tube defects NTDs in both men and mice.  Ref: 12393181 Biochim Biophys Acta, 2002
- Characterization of the platelet derived growth factor  [ **PDGFB** ]  receptor alpha  [ **PDGFRA** ]  and c kit genes in the pufferfish Fugu rubripes.  Ref: 12592706 DNA Seq, 2002
- One of the genes was the alpha subunit of the platelet derived growth factor  [ **PDGFB** ]  receptor  **Pdgfra** .  Ref: 12605401 J Neurosci Res, 2003
- In addition, treatment with RA induced expression of the platelet derived growth factor  [ **PDGFB** ]  receptor alpha  [ **PDGFRA** ] .  Ref: 12808116 Lab Invest, 2003
- We also showed that the A549 lung cancer cells expresses the platelet derived growth factor  [ **PDGFB** ]  receptor alpha  [ **PDGFRA** ] , and the inhibitory effects of Gleevec on A549 cells is likely mediated through inhibition of PDGFR alpha phosphorylation.  Ref: 12537587 Mol Cancer< MedlineTA>Mol Cancer, 2003
- This report identifies a genetic rearrangement in the eosinophilic cell line EOL 1 that results in the expression of a fusion protein comprising an N terminal region encoded by a gene of unknown function with the GenBank accession number NM\_030917 and a C terminal region derived from the intracellular domain of the platelet derived growth factor  [ **PDGFB** ]  receptor alpha  [ **PDGFRA** ]  PDGFRalpha.  Ref: 12808148 Proc Natl Acad Sci U S A, 2003
- Although bone marrow biopsies in these patients showed increased numbers of CD25 mast cells and atypical spindle shaped mast cells, patients with HES and elevated serum tryptase could be distinguished from patients with systemic mastocytosis and eosinophilia by their clinical manifestations, the absence of mast cell aggregates, the lack of a somatic KIT mutation, and the presence of the recently described fusion of the Fip1 like 1 FIP1L1 gene to the platelet derived growth factor  [ **PDGFB** ]  receptor alpha  [ **PDGFRA** ]  gene  **PDGFRA** .  Ref: 12676775 Blood, 2003
- By E11 glial precursors have begun to differentiate and at least two regions of the ventral neural tube containing glial precursor cells can be distinguished, an Nkx2.2 Neurogenin 3 Ngn3 domain and a platelet derived growth factor  [ **PDGFB** ]  receptor alpha  [ **PDGFRA** ]  PDGFRalpha Olig2 Sox10 domain.  Ref: 12237841 Glia, 2002
- We found the PLATELET DERIVED GROWTH FACTOR  [ **PDGFB** ]  RECEPTOR ALPHA  [ **PDGFRA** ] , EARLY GROWTH RESPONSE PROTEIN 1 and INSULIN LIKE GROWTH FACTOR 2 genes as well as several genes associated with MYCC and ERBB2 overexpressed by at least 2 fold in metastatic tumors in both array data sets.  Ref: 12845196 Pediatr Neurosurg, 2003
- Genetic disruption of the platelet derived growth factor  [ **PDGFB** ]  receptor alpha  [ **PDGFRA** ]  PDGFRalpha results in defects in multiple tissues in the mouse, including neural crest derivatives contributing to the frontonasal process and the aortic arch.  Ref: 12490557 Development, 2003
